# Retinitis Pigmentosa: From Pathomolecular Mechanisms to Therapeutic Strategies

**DOI:** 10.3390/medicina60010189

**Published:** 2024-01-22

**Authors:** Enzo Maria Vingolo, Simona Mascolo, Filippo Miccichè, Gregorio Manco

**Affiliations:** Sense Organs Department, UOSD of Ophtalmology, University la Sapienza of Rome, Polo Pontino-Ospedale A. Fiorini, 4019 Terracina, Italy; enzomaria.vingolo@uniroma1.it (E.M.V.); filippo.micciche@uniroma1.it (F.M.); gregorio.manco@uniroma1.it (G.M.)

**Keywords:** retinitis pigmentosa, inherited retinal disease, genotype, therapy, ERG, vitamin A, HBO, cells transplantation, AAV

## Abstract

Retinitis pigmentosa is an inherited disease, in which mutations in different types of genes lead to the death of photoreceptors and the loss of visual function. Although retinitis pigmentosa is the most common type of inherited retinal dystrophy, a clear line of therapy has not yet been defined. In this review, we will focus on the therapeutic aspect and attempt to define the advantages and disadvantages of the protocols of different therapies. The role of some therapies, such as antioxidant agents or gene therapy, has been established for years now. Many clinical trials on different genes and mutations causing RP have been conducted, and the approval of voretigene nepavorec by the FDA has been an important step forward. Nonetheless, even if gene therapy is the most promising type of treatment for these patients, other innovative strategies, such as stem cell transplantation or hyperbaric oxygen therapy, have been shown to be safe and improve visual quality during clinical trials. The treatment of this disease remains a challenge, to which we hope to find a solution as soon as possible.

## 1. Introduction

Retinitis pigmentosa is an inherited retinal neurodegenerative disease characterized by progressive photoreceptor cell death and retinal pigmented epithelium (RPE) atrophy, which initially manifests as nyctalopia, followed by continuous vision loss until blindness [1]. It is also called rod-cone dystrophy, due to the primary degeneration of rods rather than cones. Its estimated prevalence worldwide is 1/4000 [2,3]. RP can be classified according to the mode of inheritance into autosomal-dominant RP (adRP, 15–25%) [4], autosome-recessive RP (arRP, 5–20%) [5,6], and X-linked RP (x-RP, 10–15%), while in the other 40–50% cases, a pattern of inheritance cannot be detected (sporadic-RP). The age of onset and the severity of the disease in terms of visual loss depend on the mode of inheritance: adRP has the best visual acuity preservation, with the disease sometimes being detected at the age of 50; X-linked RP has the worst prognosis, with the disease starting early; and those with arRP have intermediate features [3,7]. Another way to classify RP is as syndromic, if there is the involvement of other tissues, or non-syndromic. This difference can be explained by the implication of different genes in these two types of RP. Typical RP (non-syndromic RP) may be caused by altered proteins that have a specific retinal function or are mainly expressed in the retina, whereas mutated proteins functioning in diverse cell types or tissues result in systemic manifestations (syndromic RP) [8,9]. In this review, we will focus on non-syndromic RP.

## 2. Materials and Methods

We conducted a search of the literature, including all publication years up to 1 October 2023, using MEDLINE (PubMed). The database was first searched using the following keywords: “Retinitis Pigmentosa; Inherited retinal disease; Genotype; Electroretinogram; Therapy; Vitamin A; Hyperbaric Oxygen; Cells transplantation; Adeno-associated virus”. The publication types included were reviews and clinical trials. We considered only studies in English and those with an abstract, thus reducing the count to 281 papers. The reference lists of all retrieved articles were assessed to identify additional relevant studies. The research of articles was performed using PubMed (https://pubmed.ncbi.nlm.nih.gov accessed on 1 October 2023) and Reference Citation Analysis (https://www.referencecitationanalysis.com). 

## 3. Clinical Features and Diagnosis

RP is a very disabling disease, causing the patient to lose their autonomy. The earliest symptom is nyctalopia (night blindness), explained by the primary degeneration of rods. Consequently, due to the death of an increasing number of rods, the loss of the peripheral visual field occurs, up to a few degrees of the remaining visual field around the fixation point (so-called tunnel vision). This visual acuity can be worsened by cystoid macular edema (CME), with the impairment of the central vision, and subcapsular posterior cataract (SCP), which develops in about 50% of RP patients. The mechanism implicated in these two conditions seems to be chronic low-grade intraocular inflammation [2]. This diagnosis is based on different findings. Visual acuity can be normal, especially in the first phases of RP, because, as said before, the impairment of cones generally develops later than the impairment of rods. Indirect ophthalmoscopy shows the characteristic bone spicule-like pigment changes in the periphery and/or mid-periphery due to RPE cells migrating into the retina, markedly attenuated retinal vessels, and an optic nerve with a waxy pallor; less specific are CME, vitreous cells, epiretinal membranes, and optic nerve drusen. Optical coherence tomography (OCT) is a diagnostic test able to evaluate the retinal thickness and the morphology and integrity of all the retinal layers. Typical findings are the thinning of external layers; in particular, the first layer involved is the interdigitation zone, then the ellipsoid zone (EZ), and finally the outer nuclear layer (ONL). Other common findings relate to the epiretinal membrane (ERM) and CME [10,11]. The loss of the visual field has a typical progression in RP, beginning as isolated scotomas in the mid-periphery that lead to a ring scotoma. This extends both along the outer and the inner portions of the retina, more rapidly in the first rather than the latter. But there are other patterns of visual field loss, such as the concentric one or the arcuate one, which extend from the superior periphery through the nasal or temporal retina [12]. Electroretinography (ERG) is a diagnostic test used to record the photoreceptor activity after stimulation with different types of lights. As a functional test, ERG is the key test used for the early detection and follow-up of RP, even when the patient still has no complaints of night blindness or visual field loss [3,13]. The retinal answer to the light is represented by three waves: the first, a negative wave, depends on the rods’ and cones’ answer; the positive b wave reflects the activity of bipolar cells; and the slower positive c wave represents the RPE activity. Two parameters have to be considered: the waves’ amplitude and their time to peak. Patients with RP have both reduced amplitudes of a and b waves and a prolonged time to peak [14].

## 4. Pathomolecular Mechanisms

Retinitis pigmentosa is a very heterogeneous disease. This heterogeneity depends on the great number of genes implicated and the different mutations that can hit the same gene [15]. Furthermore, family members with the same genetic mutation have different phenotypes, suggesting that unidentified genes and environmental factors can influence the RP phenotype [16]. Nowadays, more than 90 genes have been linked to RP, and this number will likely increase over the years, due to ongoing improvements in diagnostic testing techniques [17]. The first gene discovered was the RHO gene in 1990 [18], which encodes for Rhodopsine, a G protein-coupled receptor transmembrane protein. It is located on the discs of the outer segment of rods, and it is composed of a protein named rhodopsin and a chromophore named 11-cis-retinal. When stimulated by light, it activates the phototransduction cascade, which ends with the enclosure of cation channels and the beginning of the electrical pathway. Rho mutations are the most common cause of adRP, accounting for 25–30% of them [19]. PH23 is the most commonly studied mutation, with the replacement of proline with histidine in position 23. This is a gain of function mutation that leads to misfolded proteins, which are retained in the endoplasmic reticulum (ER), inducing ER stress, and finally, cell apoptosis [20,21].

The PRPH2 gene encodes for Peripherin 2 and is responsible for 5–10% of adRP cases. It is a transmembrane protein and regulates the structural integrity and function of the photoreceptor outer segment; its dysfunction leads to progressive degeneration of the photoreceptor cells [22]. Another common gene, implicated in 2% of ar-RP cases [22], is RPE65, which encodes for a visual cycle enzyme of RPE cells. It catalyzes the conversion of all-trans-retinal to photoactive 11-cis retinal, and its mutation causes the accumulation of retinoid intermediates of the visual cycle in the RPE. While for AD and AR retinitis pigmentosa, these genes cause a small percentage of all cases of the disease, the RPGR gene is the cause of 70% of XL-RP, making it a good candidate for gene therapy. Its function remains unclear, but we know it is expressed in the cilia of different tissues, including the connecting photoreceptor cilia [23].

While the genetic mutation is the trigger of the disease, a lot of molecular and cellular mechanisms act in the progression of RP. At first, a mutation in the proteins of photoreceptors can lead to cell dysfunction or to misfolded proteins, which accumulate into photoreceptors, activating te cellular stress response. The imbalance between the oxidative and antioxidative systems; the mitochondrial dysfunction, which can lead to the release of pro-apoptotic factors; the retinal remodeling, consequent to photoreceptors’ degeneration; inflammation; and the immune response are all factors that contribute to the worsening of the disease [1,23].

## 5. Therapeutical Approaches

Still, today, one of the most difficult challenges remains the treatment of patients affected by RP. Most of the pharmacological therapies discovered nowadays slow the progression of the disease, trying to improve patients’ quality of life, but none of them are curative. Some curative therapies, with the ability to slow down photoreceptors’ degeneration and preserve healthy ones, have been developed but, despite the good safety profiles they have shown and the visual improvements they have achieved, each of them presents some contraindications that mean they are not suitable for every patient. In Table 1, we summarize these therapies and their most important features.

### 5.1. Antioxidant Agents

The principal function of these agents is to prevent photoreceptors’ death and so preserve the visual acuity. One of the most important antioxidant agents in the therapy of RP is vitamin A palmitate. Vitamin A is an essential micronutrient for the normal functioning of the visual system, growth, development, and maintenance of epithelial cellular integrity, immune function, and reproduction [24]. In particular, in the retina, it is essential in the visual cycle, during which 11-cis retinal, after the activation of rhodopsin by light, is transformed in all-trans-retinal, starting with the phototransduction cascade. In 1993, Berson et al. studied the roles of vitamin A and E for RP patients, and they found that vitamin A has a protective role in the annual rate of decline in ERG amplitude compared with vitamin E and a placebo, even if significant positive effects on the visual field and the visual acuity were not observed [25]. In recent decades, the efficacy of vitamin A in the treatment of the disease has been discussed. Tiansen et al. studied the role of a low (2.5 mg) and high (102.5 mg) vitamin A palmitate diet on RHOT17M mice and RHOP347S mice. They discovered that a high dose of vitamin A preserves functional rod activity, as demonstrated by ERGs tracing in RHOT17M but not in RHOP347S mice [26]. Also, a more recent study found that excess dietary vitamin A in the presence of the RHOD190N variants exacerbates photoreceptors’ death, probably due to the accumulation of toxic bis retinoid lipofuscin fluorophores in the RPE cells, highlighting the need for genotype stratification as an inclusion criterion for further vitamin A studies [27]. We can surmise from this that interpreting the data on vitamin A palmitate in retinitis pigmentosa is subject to controversy, and recent studies have demonstrated that retinoic acid (RA) in degenerative retinal diseases is a trigger for retinal remodeling, reducing visual decline. Using RA receptor (RAR) inhibitors, such as disulfiram, can mitigate impairment of visual functions, even in mice with late-stage photoreceptor degeneration [28,29]. The role of vitamin A has also been studied in addition to other antioxidant agents. Berson et al., in 2004, showed that supplementation with vitamin A and docosahexaenoic acid (DHA) does not slow the RP progression in terms of visual acuity, visual field, and ERG [30]. DHA is an unsaturated fatty acid and it is the most common phospholipid of the retina, concentrated in the outer segment of photoreceptors [31]. Low levels of DHA in red blood cells (RBCs) have been correlated with altered ERG responses in XLRP patients, and patients with the lowest DHA-RBC concentration have the lowest cone ERG amplitudes [32]. Despite these considerations, several studies have shown that variable-DHA-dose diet supplementation does not have a significant effect either on visual acuity, visual field, or cone and rod ERG amplitude, even if the DHA-RBC concentrations are significantly higher in XLRP patients versus a placebo [33,34,35]. Berson et al. studied the combination of lutein and high doses of vitamin A, with an improvement of visual field tests in patients with a high serum lutein concentration [36]. Lutein and zeaxanthin are two dietary carotenoids that make up macular pigment in the retina, with a high antioxidant power, especially for light-generated ROS. They have been found to play a protective role in photoreceptor degeneration in rd10 mice, increasing cone and rod ERG amplitude [37,38]. Despite the long-established role of vitamin A, the data on its efficacy are controversial. It seems that the use of a combination of all these antioxidant agents has more benefits than using them separately, reducing oxidative damage of photoreceptors’ DNA and, in turn, their degeneration [39].

### 5.2. Hyperbaric Oxygen

Hyperbaric oxygen (HBO) therapy exposes the patient to a barometric pressure higher than the sea level ambient pressure, increasing the transfer of oxygen into tissues [40]. The retina is a highly metabolically active tissue, and photoreceptors are very sensitive to oxygen delivery, as demonstrated by Stone et al. [41]. According to them, this condition of transient hyperoxia may rescue retinal photoreceptors, probably by helping them to complete their metabolic requirements [41,42]. However, despite these good premises, there are only a few studies on this innovative therapy. Vingolo et al. conducted a case–control randomized clinical trial on 48 patients affected by retinitis pigmentosa. Patients were treated with 90 min HBO therapy daily, five times a week for the first month, then one week a month for the following 11 months, and finally, one week every three months for the following two years, obtaining an improvement in ERG b-wave amplitude [42]. These good results were confirmed by another study, in which HBO-treated patients showed the maintenance of visual acuity and visual field, and an increase in ERG-b wave compared to vitamin A-treated patients [43]. Nonetheless, HBO therapy is associated with some adverse effects. Repeated exposures to hyperbaric oxygen therapy led to a significant accumulation of reactive oxygen metabolites, which are already elevated in patients with RP and seem to be a principal element in the death of cones [44]. Moreover, this therapy has been associated with cataracts, keratoconus, and age-related macular degeneration (AMD) [40].

### 5.3. Stem Cell Therapy

The purpose of this type of therapy is to introduce stem cells, which can differentiate into all human cells, including retinal ones. Cell-based therapy works to replace retinal tissue with effective stem cells, restore dysfunctional cells by releasing trophic factors, and create new synapses [45]. The principal sources for these cells are represented by embryonic stem cells (ESCs) and induced pluripotent stem cells (i-PSCs). However, there are ethical concerns associated with ECSs, and they can be rejected by the immune system, while i-PSCs avoid ethical issues but may be associated with malignant characteristics. Other promising stem cells are bone-marrow-derived stem cells, including mesenchymal stem cells (MSCs) and hematopoietic stem cells (CD34+), which are multipotent cells with a reduced power of differentiation compared to pluripotent cells. In particular, MSCs have been isolated not only from bone marrow but also from adipose tissue, dental pulp, umbilical cord blood, and amniotic membrane; not only do they have the capacity to differentiate into different cells of retinal lineages but they can also secrete neurotrophic factors that enhance neural cells’ survival, differentiation, axonal outgrowth, and neural cell attachment, as well as inhibit neural cell apoptosis [46]. In 2015, Siqueira et al. conducted a clinical trial on 20 patients affected by RP, administering bone marrow stem cells with intravitreal injection. They demonstrated an improvement in vision-related quality of life after 3 months of treatment, which disappeared after 12 months [47]. The safety of autologous bone marrow MSCs’ intravitreal injection has been supported by Tuekprakhon et al. with a phase I clinical trial, in which patients experienced transient cells, flare in the anterior chamber, and IOP elevation. Furthermore, the study highlighted a high rate of IOL displacement, maybe for the traction forces near the injection site, and a single case of osseous metaplasia, which was treated efficiently with chirurgical removal [48]. The researchers also confirmed the improvement of BCVA, which returns to the baseline after 12 months. Another documented adverse effect is the onset of fibrous tissue proliferation in the vitreous cavity, which leads to tractional retinal detachment, not only after intravitreal injection [49] but also after subretinal cell implantation. Oner et al. implanted adipose-tissue-derived mesenchymal stem cells (ADMSCs) in the subretinal space of 11 RP patients, after which five out of six developed ERMs and localized peripheral retinal detachment, probably due to inadvertent preretinal injection of cells or reflux of transplanted cells from the subretinal space [50]. New methods of transplantation have been studied, in particular the delivery of stem cells into the suprachoroidal space. In this regard, the Limoli retinal restoration technique (LRRT), a variant of Pelaez’s intervention, in which the contact area between the stalk and choroid is expanded to promote paracrine autologous cell secretion into the choroidal flow, represents an important surgical advancement. The technique uses the GFs to create an environment capable of neuroenhancing the remaining retinal cells [51,52]. In a large study in the 2020s, 82 patients were treated with suprachoroidal administration of umbilical-cord-derived mesenchymal stem cells (UC-MSCs), demonstrating the safety of this therapy and the improvement in BCVA and visual field during the six months of follow-up [53]. In 2023, Ozkan et al. confirmed those results with the first suprachoroidal spheroidal mesenchymal stem cell implantation trial, which was held on 15 patients affected by RP [54].

MSCs avoid the problems related to the use of ESCs and i-PSCs, and they seem to be a promising therapy [55] due to their capacity to trans-differentiate and secrete paracrine factors. An important advantage of this therapy is its safety profile, shown in most of the clinical trials. However, important limitations are the small sample of patients and short period of follow-up, highlighting the need to continue the research in this field.

### 5.4. Gene Therapy

With gene therapy, by using different types of vectors, it is possible to bring a wild-type gene into the affected cells, promoting the expression of the working protein and suppressing the mutated one. Vectors are bio-engineered particles that inoculate, in selected cells, only a gene and some enzymes useful to its incorporation in the cells’ DNA. While that can be an effective approach, this procedure does not permit the substitution of mutated genes; for this reason, it is more effective on ar-RP or XLRP, in which the mutation causes an absence or inactivation of the protein. In ad-RP, for the dominant negative effect, the wild-type gene does not delete the toxic effect of the mutated protein, meaning these types of therapies have limited efficacy [56]. The vectors can be divided into viral, such as adeno-associated virus (AAV) or lentivirus (LV), or non-viral, such as liposomes. AAV vectors are currently the most favored for gene therapy of RP, given their ability to efficiently target various retinal layers and their excellent safety profile and low immunogenicity [57]. Instead, lentivirus-mediated gene delivery is effective at targeting RPE, but may not transduce differentiated photoreceptors efficiently, and also it can cause insertional mutagenesis [58]. Subretinal injection is the mode of gene delivery to the outer retinal layers, while with intravitreal injection, AAV transduces only the inner retinal cell layers, such as ganglion or Muller cells [59,60].

Over time, a lot of studies have been conducted to test the efficiency of AAV gene therapy, first on animal RP models and then on humans. As far as clinical trials are concerned, a phase I trial in 2016 demonstrated the safety of the subretinal administration of rAAV2-VMD2-hMERTK in six participants with MERTK-associated RP, but with only one patient maintaining the visual gain at the 2-year follow up [61]. Most recently, Kapetanovic et al. reported the first phase I/II dose escalation clinical trial for X-linked RP caused by mutations in the RP GTPase regulator. A cohort of 18 patients was treated with subretinal delivery of an adeno-associated viral vector encoding codon-optimized human RPGR (AAV8.coRPGR). This study showed that AAV8.coPRGR did not have any dose-limiting toxicities and, in some patients, it restored the loss of the visual field [62]. The results of this trial have been compared with untreated eyes, demonstrating the efficacy of cotoretigene toliparvovec (BIIB112/AAV8-RPGR) at 12 months after injection [63]. In particular, four treated patients, who received the four highest doses of therapy, versus a single untreated one, showed early and sustained improvements in visual function over 12 months.

An important step forward in gene therapy was the first phase III clinical trial, conducted by Russel et al. in 2017, on voretigene neparvovec (AAV2-hRPE65v2) in patients affected by inherited retinal disease due to RPE-65 mutation, demonstrating an improvement of light sensibility, BCVA, and visual field [64]. RPE-65 encodes for all-trans retinal ester isomerase, implicated in the visual cycle. The wild-type RPE produces 11-cis retinal, which is transported on the outer segment membrane of photoreceptors’ cells, initiating the phototransduction cascade after light exposure. This study promoted the approval of Luxturna (voretigene neparvovec) by the US Food and Drug Administration in gene therapy for Leber congenital amaurosis (LCA).

So, gene therapy is the most promising therapeutic approach for RP, but it has two important limitations. First of all, the heterogeneity of gene mutations in RP makes genetically targeted treatment difficult to develop. Also, gene augmentation strategies for RP will only be effective if the photoreceptors are still present, even if they are sickly. As such, in order to attain the maximal therapeutic effect, the timing of the intervention is of the essence [58].

### 5.5. New Prospective Therapies

As said before, gene therapy can offer a useful strategy for ar-RP, while it is less effective for ad-RP. Lately, a new system derived from bacteria or archaea has been investigated, which is based on the joint action of the clustered regularly interspaced short palindromic repeat (CRISPR) and Cas9, creating an RNA-guided DNA cleavage system. Once the Cas9 nuclease cleaves double-stranded DNA in a site-specific manner, DNA repair machinery is activated, swapping out the mutant with the wild-type sequence [65]. Some studies in vivo have already tested the efficacy of this type of therapy [66,67]. With the CRISPR/Cas9 approach, it is possible to target multiple genes simultaneously, avoiding the problem related to the heterogeneity of the disease. Another recent type of therapy is optogenetics, in which photosensitive optic proteins, introduced with vectors into the retina, can be expressed in damaged cell membranes, resynthesizing degenerated photoreceptors, and can confer photosensitive ability to residual retinal cells [1,68]. However, when the disease is too advanced, the substrate for all these techniques is missing, and retinal prostheses are the only possible choice. With these devices, it is possible to convert the light stimuli into an electrical signal, which stimulates the remaining structures of the retina, activating the optic nerve and transmitting the images to the brain. This conversion happens thanks to an epiretinal, subretinal, or suprachoroidal microarray, which directly or indirectly stimulates retinal ganglion cells [69]. At the moment, we only know two prostheses, Argus II and Alpha-IMS, with only the first one approved by the FDA. Furthermore, serious adverse effects have been reported with this approach, such as conjunctival erosions, hypotony, explantation, and retinal detachment [70].

## 6. Conclusions

Retinitis pigmentosa, like other inherited retinal diseases, remains an unstoppable disease today, leading the patient to become totally blind. Despite the scientific and technological advances, there is currently no curative treatment for RP, with only a small group with confirmed RPE65 mutations being eligible to receive the approved gene therapy (voretigene neparvovec). The current therapeutic armamentarium is limited to vitamin A supplements, protection from sunlight, avoidance of smoking, enhancing physical activity, avoiding obesity, visual aids, and medical and surgical interventions to treat ophthalmic comorbidities (cataract surgery for posterior subcapsular cataract and intraocular corticosteroid implant for cystoid macular edema), all aiming to slow down the disease’s progression. Despite the long-established role of vitamin A palmitate and its low cost, it remains a noncurative therapy, with the only purpose of its use being to slow down the disease’s progression. Furthermore, interpreting data from clinical trials on this therapy is subject to controversy, highlighting the need to genotype the patients who could be of most benefit [27]. The ethical and immunological problems related to stem cell transplantation have been bypassed by intravitreal, subretinal, and suprachoroidal MSCs implantation, which has shown a good safety profile and promising results. However, our expectations related to this therapy are based on limited clinical data, which could be strengthened by expanding the sample of patients and extending the follow-up period of future clinical trials. Hyperbaric oxygen therapy, meanwhile, has shown positive preliminary results but requires more robust evidence, and we hope, especially for its easy administration, that this type of therapy in the future will be further investigated. The heterogeneity of the disease, the need to genotype the patients, the limited duration of positive effects, the need to have residual photoreceptors, and the cost of therapy are the key limitations associated with gene therapy. To avoid these problems, researchers could work to develop systems that can rapidly genotype all patients, along with biomarkers to identify patients in earlier disease stages, before significant photoreceptor loss occurs. Continued multidisciplinary research efforts to bypass current therapies’ limitations and to develop new curative therapies that could halt vision loss in RP patients are of vital importance. Combining approaches may be necessary to comprehensively treat this complex and heterogeneous condition.

## Figures and Tables

**Table 1:** Therapeutic approaches in retinitis pigmentosa.

Type of Therapy	Mechanism of Action	Advantages	Disadvantages
Vitamin A Palmitate	The principal element of the visual cycle.	Easy administration, low cost.	Conflicting results in clinical trials, low effectiveness, teratogenic in high dose.
Stem Cell Therapy	Replace dysfunctional retinal cells and release neurotrophic and growth factors.	Good safety profile in particular for MSCs, improvement in visual quality.	Ethical issues for ESCs, malignant growth for iPSCs, difficulty handling cells, clinical trials with small samples of patients and short follow-up period.
Hyperbaric Oxygen	Increase the transfer of oxygen into tissues, helping the retinal cells with their metabolic request.	Easy administration, improvement in amplitude ERG b wave.	Few trials, accumulation of radical oxygen species (ROS), high rate of adverse events.
Gene Therapy	Replace the mutated gene with the wild-type.	Many trials both in animal models and humans, good safety profile, good results in clinical trials.	Heterogeneity of the disease, necessity to genotype the patient, non-advanced-stage disease, limited duration of positive effect.

## Data Availability

The original contributions presented in the study are included in the article, further inquiries can be directed to the corresponding author.

## References

[1] Liu W., Liu S., Li P., Yao K. (2022). Retinitis Pigmentosa: Progress in Molecular Pathology and Biotherapeutical Strategies. Int. J. Mol. Sci..

[2] Natarajan S. (2011). Retinitis pigmentosa: A brief overview. Indian J. Ophthalmol..

[3] Hamel C. (2006). Retinitis pigmentosa. Orphanet J. Rare Dis..

[4] Jordan S.A., Farrar G.J., Kenna P., Humphries M.M., Sheils D.M., Kumar-Singh R., Sharp E.M., Soriano N., Ayuso C., Benitez J. (1993). Localization of an autosomal dominant retinitis pigmentosa gene to chromosome 7q. Nat. Genet..

[5] Banerjee P., Kleyn P.W., Knowles J.A., Lewis C.A., Ross B.M., Parano E., Kovats S.G., Lee J.J., Penchaszadeh G.K., Ott J. (1998). TULP1 mutation in two extended Dominican kindreds with autosomal recessive retinitis pigmentosa. Nat. Genet..

[6] Maw M.A., Kennedy B., Knight A., Bridges R., Roth K.E., Mani E.J., Mukkadan J.K., Nancarrow D., Crabb J.W., Denton M.J. (1997). Mutation of the gene encoding cellular retinaldehyde-binding protein in autosomal recessive retinitis pigmentosa. Nat. Genet..

[7] Fishman G.A. (1978). Retinitis pigmentosa. Vis. Loss. Arch. Ophthalmol..

[8] Waters A.M., Beales P.L. (2011). Ciliopathies: An expanding disease spectrum. Pediatr. Nephrol..

[9] Wheway G., Parry D.A., Johnson C.A. (2014). The role of primary cilia in the development and disease of the retina. Organogenesis.

[10] Malvasi M., Casillo L., Avogaro F., Abbouda A., Vingolo E.M. (2023). Gene Therapy in Hereditary Retinal Dystrophies: The Usefulness of Diagnostic Tools in Candidate Patient Selections. Int. J. Mol. Sci..

[11] Liu G., Liu X., Li H., Du Q., Wang F. (2016). Optical coherence tomographic analysis of retina in retinitis pigmentosa patients. Ophthalmic Res..

[12] Grover S., Fishman G.A., Brown J. (1998). Patterns of visual field progression in patients with retinitis pigmentosa. Ophthalmology.

[13] Ebdali S., Hashemi B., Hashemi H., Jafarzadehpur E., Asgari S. (2018). Time and frequency components of ERG responses in retinitis pigmentosa. Int. Ophthalmol..

[14] Hartong D.T., Berson E.L., Dryja T.P. (2006). Retinitis pigmentosa. Lancet.

[15] Daiger S.P., Sullivan L.S., Bowne S.J. (2013). Genes and mutations causing retinitis pigmentosa. Clin. Genet..

[16] Verbakel S.K., van Huet R.A.C., Boon C.J.F., den Hollander A.I., Collin R.W.J., Klaver C.C.W., Hoyng C.B., Roepman R., Klevering B.J. (2018). Non-syndromic retinitis pigmentosa. Prog. Retin. Eye Res..

[17] Nguyen X.T., Moekotte L., Plomp A.S., Bergen A.A., van Genderen M.M., Boon C.J.F. (2023). Retinitis Pigmentosa: Current Clinical Management and Emerging Therapies. Int. J. Mol. Sci..

[18] Dryja T.P., McGee T.L., Reichel E., Hahn L.B., Cowley G.S., Yandell D.W., Sandberg M.A., Berson E.L. (1990). A point mutation of the rhodopsin gene in one form of retinitis pigmentosa. Nature.

[19] Yan Z., Yao Y., Li L., Cai L., Zhang H., Zhang S., Xiao Q., Wang X., Zuo E., Xu C. (2023). Treatment of autosomal dominant retinitis pigmentosa caused by RHO-P23H mutation with high-fidelity Cas13X in mice. Mol. Ther. Nucleic Acids.

[20] Illing M.E., Rajan R.S., Bence N.F., Kopito R.R. (2002). A rhodopsin mutant linked to autosomal dominant retinitis pigmentosa is prone to aggregate and interacts with the ubiquitin proteasome system. J. Biol. Chem..

[21] Tam B.M., Moritz O.L. (2006). Characterization of rhodopsin P23H-induced retinal degeneration in a Xenopus laevis model of retinitis pigmentosa. Investig. Ophthalmol. Vis. Sci..

[22] Arikawa K., Molday L.L., Molday R.S., Williams D.S. (1992). Localization of peripherin/rds in the disk membranes of cone and rod photoreceptors: Relationship to disk membrane morphogenesis and retinal degeneration. J. Cell Biol..

[23] Becherucci V., Bacci G.M., Marziali E., Sodi A., Bambi F., Caputo R. (2023). The New Era of Therapeutic Strategies for the Treatment of Retinitis Pigmentosa: A Narrative Review of Pathomolecular Mechanisms for the Development of Cell-Based Therapies. Biomedicines.

[24] Bennasir H., Sridhar S., Abdel-Razek T.T. (2010). Vitamin A from physiology to disease prevention. Research in Autism Spectrum Disorders. Int. J. Pharm. Sci. Rev..

[25] Berson E.L., Rosner B., Sandberg M.A., Hayes K.C., Nicholson B.W., Weigel-DiFranco C., Willett W. (1993). A randomized trial of vitamin A and vitamin E supplementation for retinitis pigmentosa. Arch. Ophthalmol..

[26] Li T., Sandberg M.A., Pawlyk B.S., Rosner B., Hayes K.C., Dryja T.P., Berson E.L. (1998). Effect of vitamin A supplementation on rhodopsin mutants threonine-17 --> methionine and proline-347 --> serine in transgenic mice and in cell cultures. Proc. Natl. Acad. Sci. USA.

[27] Cui X., Kim H.J., Cheng C.H., Jenny L.A., Lima de Carvalho J.R., Chang Y.J., Kong Y., Hsu C.W., Huang I.W., Ragi S.D. (2022). Long-term vitamin A supplementation in a preclinical mouse model for RhoD190N-associated retinitis pigmentosa. Hum. Mol. Genet..

[28] Telias M., Sit K.K., Frozenfar D., Smith B., Misra A., Goard M.J., Kramer R.H. (2022). Retinoic acid inhibitors mitigate vision loss in a mouse model of retinal degeneration. Sci. Adv..

[29] Telias M., Denlinger B., Helft Z., Thornton C., Beckwith-Cohen B., Kramer R.H. (2019). Retinoic Acid Induces Hyperactivity, and Blocking Its Receptor Unmasks Light Responses and Augments Vision in Retinal Degeneration. Neuron.

[30] Berson E.L., Rosner B., Sandberg M.A., Weigel-DiFranco C., Moser A., Brockhurst R.J., Hayes K.C., Johnson C.A., Anderson E.J., Gaudio A.R. (2004). Clinical trial of docosahexaenoic acid in patients with retinitis pigmentosa receiving vitamin A treatment. Arch. Ophthalmol..

[31] Fliesler S.J., Anderson R.E. (1983). Chemistry and metabolism of lipids in the vertebrate retina. Prog. Lipid Res..

[32] Hoffman D.R., Birch D.G. (1995). Docosahexaenoic acid in red blood cells of patients with X-linked retinitis pigmentosa. Investig. Ophthalmol. Vis. Sci..

[33] Hoffman D.R., Locke K.G., Wheaton D.H., Fish G.E., Spencer R., Birch D.G. (2004). A randomized, placebo-controlled clinical trial of docosahexaenoic acid supplementation for X-linked retinitis pigmentosa. Am. J. Ophthalmol..

[34] Hoffman D.R., Hughbanks-Wheaton D.K., Pearson N.S., Fish G.E., Spencer R., Takacs A., Klein M., Locke K.G., Birch D.G. (2014). Four-year placebo-controlled trial of docosahexaenoic acid in X-linked retinitis pigmentosa (DHAX Trial): A randomized clinical trial. JAMA Ophthalmol..

[35] Hoffman D.R., Hughbanks-Wheaton D.K., Spencer R., Fish G.E., Pearson N.S., Wang Y.Z., Klein M., Takacs A., Locke K.G., Birch D.G. (2015). Docosahexaenoic Acid Slows Visual Field Progression in X-Linked Retinitis Pigmentosa: Ancillary Outcomes of the DHAX Trial. Investig. Ophthalmol. Vis. Sci..

[36] Berson E.L., Rosner B., Sandberg M.A., Weigel-DiFranco C., Brockhurst R.J., Hayes K.C., Johnson E.J., Anderson E.J., Johnson C.A., Gaudio A.R. (2010). Clinical trial of lutein in patients with retinitis pigmentosa receiving vitamin A. Arch. Ophthalmol..

[37] Zhang H.J., Liu X.B., Chen X.M., Kong Q.H., Liu Y.S., So K.F., Chen J.S., Xu Y., Mi X.S., Tang S.B. (2022). Lutein delays photoreceptor degeneration in a mouse model of retinitis pigmentosa. Neural. Regen. Res..

[38] Yu M., Yan W., Beight C. (2018). Lutein and Zeaxanthin Isomers Reduce Photoreceptor Degeneration in the Pde6brd10 Mouse Model of Retinitis Pigmentosa. Biomed. Res. Int..

[39] Sanz M.M., Johnson L.E., Ahuja S., Ekström P.A., Romero J., van Veen T. (2007). Significant photoreceptor rescue by treatment with a combination of antioxidants in an animal model for retinal degeneration. Neuroscience.

[40] McMonnies C.W. (2015). Hyperbaric oxygen therapy and the possibility of ocular complications or contraindications. Clin. Exp. Optom..

[41] Stone J., Maslim J., Valter K., Mervin K., Anderson R.E. (1996). The influence of oxygen levels on the death and survival of photoreceptors. Retinal Degeneration in Degenerative Diseases of the Retina.

[42] Vingolo E.M., Pelaia P., Forte R., Rocco M., Giusti C., Rispoli E. (1998). Does hyperbaric oxygen (HBO) delivery rescue retinal photoreceptors in retinitis pigmentosa?. Doc. Ophthalmol..

[43] Vingolo E.M., Rocco M., Grenga P., Salvatore S., Pelaia P. (2008). Slowing the degenerative process, long lasting effect of hyperbaric oxygen therapy in retinitis pigmentosa. Graefe’s Arch. Clin. Exp. Ophthalmol..

[44] Song D.J., Bao X.L., Fan B., Li G.Y. (2023). Mechanism of Cone Degeneration in Retinitis Pigmentosa. Cell. Mol. Neurobiol..

[45] He Y., Zhang Y., Liu X., Ghazaryan E., Li Y., Xie J., Su G. (2014). Recent advances of stem cell therapy for retinitis pigmentosa. Int. J. Mol. Sci..

[46] Florido A., Vingolo E.M., Limoli P., Contento L. (2021). Mesenchymal Stem Cells for Treatment of Retinitis Pigmentosa: Short Review. J. Stem Cells Res. Dev. Ther..

[47] Siqueira R.C., Messias A., Voltarelli J.C., Scott I.U., Jorge R. (2011). Intravitreal injection of autologous bone marrow-derived mononuclear cells for hereditary retinal dystrophy: A phase I trial. Retina.

[48] Tuekprakhon A., Sangkitporn S., Trinavarat A., Pawestri A.R., Vamvanij V., Ruangchainikom M., Luksanapruksa P., Pongpaksupasin P., Khorchai A., Dambua A. (2021). Intravitreal autologous mesenchymal stem cell transplantation: A non-randomized phase I clinical trial in patients with retinitis pigmentosa. Stem Cell Res. Ther..

[49] Satarian L., Nourinia R., Safi S., Kanavi M.R., Jarughi N., Daftarian N., Arab L., Aghdami N., Ahmadieh H., Baharvand H. (2017). Intravitreal Injection of Bone Marrow Mesenchymal Stem Cells in Patients with Advanced Retinitis Pigmentosa; a Safety Study. J. Ophthalmic Vis. Res..

[50] Oner A., Gonen Z.B., Sinim N., Cetin M., Ozkul Y. (2016). Subretinal adipose tissue-derived mesenchymal stem cell implantation in advanced stage retinitis pigmentosa: A phase I clinical safety study. Stem Cell Res. Ther..

[51] Limoli P.G., Vingolo E.M., Limoli C., Scalinci S.Z., Nebbioso M. (2018). Regenerative therapy by suprachoroidal cell autograft in dry age-related macular degeneration: Preliminary in vivo report. J. Vis. Exp..

[52] Limoli P.G., Vingolo E.M., Limoli C., Nebbioso M. (2019). Stem Cell Surgery and Growth Factors in Retinitis Pigmentosa Patients: Pilot Study after Literature Review. Biomedicines.

[53] Kahraman N.S., Oner A. (2020). Umbilical cord derived mesenchymal stem cell implantation in retinitis pigmentosa: A 6-month follow-up results of a phase 3 trial. Int. J. Ophthalmol..

[54] Özkan B., Yılmaz Tuğan B., Hemşinlioğlu C., Sır Karakuş G., Şahin Ö., Ovalı E. (2023). Suprachoroidal spheroidal mesenchymal stem cell implantation in retinitis pigmentosa: Clinical results of 6 months follow-up. Stem Cell Res. Ther..

[55] Sharma A., Jaganathan B.G. (2021). Stem Cell Therapy for Retinal Degeneration: The Evidence to Date. Biologics.

[56] Petrs-Silva H., Linden R. (2014). Advances in gene therapy technologies to treat retinitis pigmentosa. Clin. Ophthalmol..

[57] Trapani I., Puppo A., Auricchio A. (2014). Vector platforms for gene therapy of inherited retinopathies. Prog. Retin. Eye Res..

[58] Bennett J. (2017). Taking Stock of Retinal Gene Therapy: Looking Back and Moving Forward. Mol. Ther..

[59] Dudus L., Anand V., Acland G.M., Chen S.-J., Wilson J.M., Fisher K.J., Maguire A.M., Bennett J. (1999). Persistent transgene product in retina, optic nerve and brain after intraocular injection of rAAV. Vis. Res..

[60] Harvey A.R., Kamphuis W., Eggers R., Symons N.A., Blits B., Niclou S., Boer G.J., Verhaagen J. (2002). Intravitreal injection of adeno-associated viral vectors results in the transduction of different types of retinal neurons in neonatal and adult rats: A comparison with lentiviral vectors. Mol. Cell. Neurosci..

[61] Ghazi N.G., Abboud E.B., Nowilaty S.R., Alkuraya H., Alhommadi A., Cai H., Hou R., Deng W.-T., Boye S.L., Almaghamsi A. (2016). Treatment of retinitis pigmentosa due to MERTK mutations by ocular subretinal injection of adeno-associated virus gene vector: Results of a phase I trial. Hum. Genet..

[62] Cehajic-Kapetanovic J., Xue K., Martinez-Fernandez de la Camara C., Nanda A., Davies A., Wood L.J., Salvetti A.P., Fischer M.D., Aylward J.W., Barnard A.R. (2020). Initial results from a first-in-human gene therapy trial on X-linked retinitis pigmentosa caused by mutations in RPGR. Nat. Med..

[63] von Krusenstiern L., Liu J., Liao E., Gow J.A., Chen G., Ong T., Lotery A.J., Jalil A., Lam B.L., MacLaren R.E. (2023). Changes in Retinal Sensitivity Associated With Cotoretigene Toliparvovec in X-Linked Retinitis Pigmentosa With RPGR Gene Variations. JAMA Ophthalmol..

[64] Russell S., Bennett J., Wellman J.A., Chung D.C., Yu Z.-F., Tillman A., Wittes J., Pappas J., Elci O., McCague S. (2017). Efficacy and safety of voretigene neparvovec (AAV2-hRPE65v2) in patients with RPE65 -mediated inherited retinal dystrophy: A randomised, controlled, open-label, phase 3 trial. Lancet.

[65] Dias M.F., Joo K., Kemp J.A., Fialho S.L., da Silva Cunha A., Woo S.J., Kwon Y.J. (2018). Molecular genetics and emerging therapies for retinitis pigmentosa: Basic research and clinical perspectives. Prog. Retin. Eye Res..

[66] Gumerson J.D., Alsufyani A., Yu W., Lei J., Sun X., Dong L., Wu Z., Li T. (2022). Restoration of RPGR expression in vivo using CRISPR/Cas9 gene editing. Gene Ther..

[67] Xi Z., Vats A., Sahel J.A., Chen Y., Byrne L.C. (2022). Gene augmentation prevents retinal degeneration in a CRISPR/Cas9-based mouse model of PRPF31 retinitis pigmentosa. Nat. Commun..

[68] Simunovic M.P., Shen W., Lin J.Y., Protti D.A., Lisowski L., Gillies M.C. (2019). Optogenetic approaches to vision restoration. Exp. Eye Res..

[69] Wu K.Y., Mina M., Sahyoun J.Y., Kalevar A., Tran S.D. (2023). Retinal Prostheses: Engineering and Clinical Perspectives for Vision Restoration. Sensors.

[70] Stanga P.E., Tsamis E., Siso-Fuertes I., Dorn J.D., Merlini F., Fisher A., Crawford F.I., Kasbia S.S., Papayannis A., Baseler H.A. (2021). Electronic Retinal Prosthesis for Severe Loss of Vision in Geographic Atrophy in Age-Related Macular Degeneration: First-in-Human Use. Eur. J. Ophthalmol..

